# Transcatheter Embolization of Ruptured Uterine Artery Pseudoaneurysm: A Case Report

**DOI:** 10.31729/jnma.4989

**Published:** 2020-06

**Authors:** Sristi Singh, Lochan Shrestha, Sagun Manandhar, Pooja Roy

**Affiliations:** 1 Interventional Radiology Unit, Department of Radiology and Imaging, Patan Academy of Health Sciences, Lalitpur, Nepal

**Keywords:** *Nepal*, *postpartum hemorrhage*, *pseudoaneurysm*, *uterine artery embolizatio*

## Abstract

A 30-years-old post-cesarean lady presented with a secondary post-partum hemorrhage for a week, complicated by anemia, which required blood transfusion. Despite conservative medical management, the bleeding persisted and ultrasonography demonstrated a ruptured left uterine artery pseudoaneurysm. Computed Tomography angiogram was performed for confirmation and planning for embolization. Transcatheter directed uterine artery pseudoaneurysm embolization was performed. Her bleeding was controlled with an uneventful post-procedure period and was discharged after two days. This case report summarizes the procedure of transcatheter embolization of uterine artery pseudoaneurysm in a tertiary care hospital in Nepal.

## INTRODUCTION

Pseudoaneurysm of the uterine artery is a potentially life-threatening^1,2^ rare cause of delayed postpartum hemorrhage (PPH).^3^The risk of its rupture is proportionate to the size and intramural pressure.^4^ Transabdominal Doppler ultrasonography and angiography are the investigations for its diagnosis.^4^ Transcatheter uterine artery embolization (UAE) is a successful technique for managing vascular-related uterine hemorrhages, including that from uterine artery pseudoaneurysm. Despite its advantages, it is not widely used due to lack of expertise in developing countries like Nepal.^5^

We report a case of uterine artery pseudoaneurysm presenting with secondary PPH and managed successfully with UAE.

## CASE REPORT

A 30-years-old P2L2 lady was referred to our hospital 40 days post-partum, with complaints of excessive intermittent per vaginal bleeding for a week. At term, she had undergone an emergency lower section caesarian section (LSCS), for thick meconium-stained liquor, with an uneventful peripartum period, and delivered a healthy

live baby. On examination, she was pale even after transfusion of two pints of whole blood in a previous health care center for low hemoglobin levels. She had a well-healed Pfannenstiel scar with the soft and non-tender abdomen. Her per-vaginal examination revealed approximately 8-weeks sized contracted uterus and blood in the cervix without evidence of active bleeding on per speculum examination.

**Figure 1:**
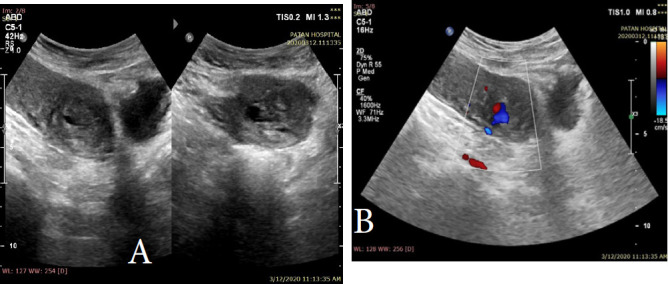
(A) 2D ultrasonography shows heterogeneous hypoechoic area in the lower uterine segment suggestive of hematoma. (B) Color Doppler shows turbulent flow (ying-yang sign) indicating pseudoaneurysm.

Hematological and biochemical parameters were done, which showed hemoglobin of 9.1gm/dl. Transabdominal ultrasonography demonstrated a ruptured uterine artery pseudoaneurysm with surrounding hematoma as a heterogeneous hypoechoic lesion at the lower uterine segment with the turbulent bidirectional flow with characteristic ying-yang appearance on color Doppler, and to-and-fro pattern on pulsed Doppler. The left uterine artery was seen extending into this site with a focal area of dilatation representing its supplying artery (Figure 1).

Pelvic computed tomography (CT) angiography with non-ionic contrast confirmed the diagnosis with well-defined, contrast filled pseudoaneurysm arising from the left uterine artery. No thrombus was identified within it. Although surrounding hematoma was noted, there was no evidence of active bleed.

**Figure 2:**
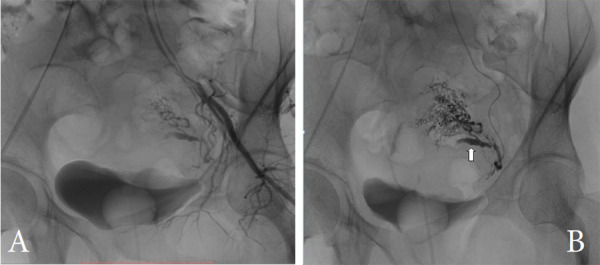
(A) Selective angiogram of the anterior division of the left anterior iliac artery showing tortuous, dilated left uterine artery. (B) Super selective angiogram of the left uterine artery shows pseudoaneurysm (arrow) with a distal hypervascular parenchymal blush.

After a discussion with Obstetrics and Gynecology unit, the patient was transferred to the angiography suite for UAE. The contralateral femoral artery was accessed under ultrasound guidance. Due to limited hardware and resources, the procedure was started with 4 Fr Cobra catheter (Terumo) and Terumo glide wire. Left iliac angiogram demonstrated the pseudoaneurysm arising from a uterine branch of the left internal iliac artery. Procreate microcatheter of 2.4 Fr was then used for super-selective cannulation of the tortuous uterine artery (Figure 2), but navigation beyond the aneurysm was not possible since the Cobra catheter was unable to provide enough support.

**Figure 3:**
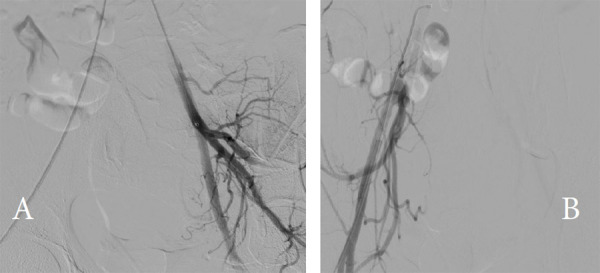
(A) Post embolization angiogram showing complete occlusion of pseudoaneurysm and parenchyma blush. (B) Check angiogram through the right iliac artery shows no contralateral flow.

An impromptu decision to use the Judkins Right (JR) guiding catheter was made because it was readily available since the suite was also shared for cardiac interventions. Therefore, a 5 Fr JR catheter in co-axial with the Progreate microcatheter was used to inject slurred gel foam to embolize the hypervascular collaterals, followed by Polyvinyl Alcohol (PVA) particles of 300-400μm size for complete and permanent embolization of the pseudoaneurysm. Complete obliteration of the pseudoaneurysm was demonstrated in immediate post-embolization angiogram images (Figure 3).

**Figure 4:**
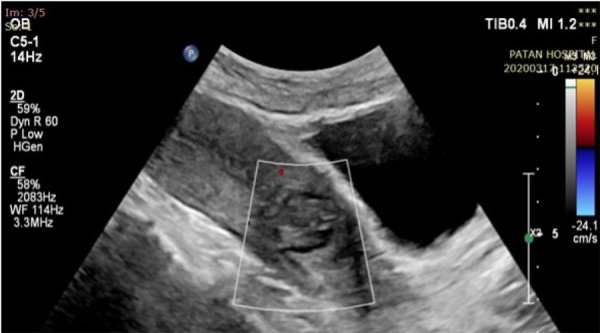
Post embolization Doppler ultrasonography shows the absence of turbulent vascularity of pseudoaneurysm.

The post-procedure period was uneventful, and per-vaginal bleeding resolved. The patient was comfortable and was observed for two days. Post-procedural follow-up transabdominal ultrasonography with color and spectral Doppler on day three revealed no blood flow at the previous aneurysm site (Figure 4).

The patient was then discharged and advised for follow up after 6 months or sooner if any bleeding reoccurs.

## DISCUSSION

Retained products of conception, endometritis, placental bed subinvolution, pseudoaneurysm of the uterine artery, arteriovenous malformations, and choriocarcinoma are some of the causes of PPH, which is also a leading factor for increased maternal morbidity and mortality in developing countries including Nepal.^6^ Uterine artery pseudoaneurysm should be considered in all cases of massive vaginal bleeding occurring after Obstetric and Gynecological procedures.

Pseudoaneurysm of the uterine artery causes delayed postpartum hemorrhage, which may be misdiagnosed as retained products of conception, but it can effortlessly be diagnosed with initial non-invasive diagnostic modalities, like ultrasonography with Doppler and contrast-enhanced computed tomography, avoiding unnecessary curettage leading to life-threatening blood loss.^6^ Furthermore, based on the fact that bleeding can resolve spontaneously and only to recur at a later stage, such imaging tests should be undertaken to rule out pseudoaneurysm before discharging patients.

Catheter angiography remains the gold standard in diagnosing pseudoaneurysms, and UAE is effective as well as safe to treat post-partum hemorrhage, preserving the reproductive function of the patient.^7^ Life-threatening bleeding can occur spontaneously or after provocation, such as intercourse; therefore, embolization of the pseudoaneurysm should be performed when the diagnosis is made.^6^

Vascular interventions are performed only in few centers within the capital of Nepal. Furthermore, we have faced infrastructural and logistical challenges, including scarcity of appropriate hardware. Overcoming such obstacles require loco-regional development of technologies,^5^ but until then, we must find innovative and workaround methods.^8^

Learning points from this case are consideration of a potentially life-threatening condition of ruptured uterine artery pseudoaneurysm in women presenting with vaginal bleeding after Obstetric and Gynecological procedures and UAE as a viable effective treatment measure in such cases before resorting to surgery.

**Consent: JNMA** Case Report Consent Form was signed by the patient and the original article is attached to the patient’s chart.

## Conflict of Interest:

**None.**
